# How to Subscribe

**Published:** 1861-12

**Authors:** 


### HOW TO SUBSCRIBE.

Send your letter to the simple address of Dr. W. W. Hall, New-York. But before you send it, attach a dollar bill with a pin to the top of your letter-sheet; *then* under that write your name in full, with the name of State, county, and post-office; seal the letter with a wafer, and put it in the post-office yourself without saying any thing to any body. By so doing, you will not have to write another letter to say that you had forgotten to inclose the all-important dollar. It is quite certain that the troublous times have made some of our subscribers too poor to take our JOURNAL longer; to such we will continue to send it, if they forward the names of two new subscribers, and two dollars. To any clergyman who will send us two new subscribers with two dollars, we will send a third number for their trouble. This is done because, in too many cases, their already scant salaries have been scaled down to a point so low as to shame the churches. To any person who will send three dollars, with three new subscribers' names, the fourth copy will be sent for their trouble.

We have never offered to pay persons for taking the JOURNAL, in portraits, paintings, chances, or any thing else, nor will we "club" with any other publication, nor do we want any one to take it who does not consider it worth a dollar. We know there are some who value it far above the subscription-price, but who do not feel as if they could spare the money, and yet not only want the JOURNAL for next year, but would like very much to have one or more of our other publications; to such and to all others we offer any of our dollar volumes for three new subscribers, and any one of our dollar and a quarter volumes for four new subscribers, or "Soldier Health" for one new subscriber. If any of these volumes are requested to be sent by mail, sixteen cents must be sent to pay postage.

To any one who will send twenty new subscribers at one time, we will give, at our office, the eight bound volumes of HALL'S JOURNAL OF HEALTH. To any one sending thirty-four new subscribers, we will give a copy of each one of our publications, which are as follows:

| | |
|---|---|
| Eight bound vols. HALL'S JOUR. HEALTH, each | $1.25 |
| Vols. 1 and 2 FIRESIDE MONTHLY, each . | 1.25 |
| BRONCHITIS AND KINDRED DISEASES, . | 1.00 |
| CONSUMPTION, . . . . . . . . | 1.00 |
| HEALTH AND DISEASE, . . . . | 1.00 |
| SLEEP, . . . . . . . . . | 1.25 |
| SOLDIER HEALTH, . . . . . . | 25 |

Or seventeen dollars' worth of books for thirty-four new subscribers. In other words, we offer half a dollar's worth of books for the trouble of getting one new subscriber. In a

SINGLE DAY

An active young person could earn the whole set. Could two or three

YOUNG LADIES

spend a bright, bracing winter's morning in a better way, than by turning out with a resolve to obtain subscribers enough to enable them to present to their minister the whole fifteen volumes? In fact, we so heartily admire a kindly feeling toward a minister, on the part of the young of his charge, and so truly delight to see activity, energy, and enterprise in youth, that we hereby

PROMISE

to give the whole fifteen volumes, and five dollars in money besides, to any

YOUNG LADY

who, on or before Christmas-day next, shall send us thirty-four new subscribers, for the purpose of presenting the books to a clergyman.

In all cases, sums *under* one dollar should be sent in postage-stamps, and *over* five dollars in a draft payable to our order.

